# Advanced fractionation process for wine-based products diversification

**DOI:** 10.1007/s13197-020-04957-7

**Published:** 2021-01-18

**Authors:** Gabriele Di Giacomo, Pietro Romano

**Affiliations:** Department of Industrial and Information Engineering and Economics, University of L’Aquila, Via Giovanni Gronchi, n. 18, 67100 L’Aquila, Italy

**Keywords:** Industrial wine, Dealcoholized wine, Solid extract, Fractionation process

## Abstract

Wine fractionation is an old practice widely applied for many reasons, including the production of food-grade alcohol and spirits, alcohol-reduced wines and beverages, functional products, and aromas. The purpose is the need to satisfy different lifestyles and legal constraints. The raw material, usually called industrial wine, includes wine overproduction and wine not used as such: mainly table wine, the fermented juice of unsold table grapes, and quality wine. Three technologies are currently in use: Vacuum distillation, Reverse osmosis in dialyzing mode, and the Spinning cone column. The process developed in this work results from the integration of a multistage reverse osmosis section operating in dialyzing mode, with the Atmospheric distillation of the permeate stream; the two most applied technologies for fractionating liquid mixtures. This process allows the fractionation of the wine into four products (the vegetation water, the azeotropic Ethanol, a concentrated aqueous solution of the solid extract, and a concentrated alcoholic solution of volatile aroma compounds) while preserving sensorial, nutritional and functional properties of the individual compounds. Then, the proper recombination of these products gives rise to a wide variety of wine-based products to meet the specifications of each market segment. The process is environmentally friendly and, in comparison with the competitors, is less energy-intensive, other than resilient and flexible regarding the production potentiality.

## Introduction

The world wine (fermented juice of grapes) production in the last decade lies in the range 26–29 billion litres per year, with a constant global overproduction of about 15%, and involves all the continents with the exclusion of Antarctica. More than 60% of this enormous amount is produced in Europe, mostly by Italy, France, and Spain (OIV Statistical Report 2019). European wine is consumed locally and exported, mainly in the USA, but there is more than 15% of overproduction, despite the recent “Wine Reform” (Agreement between the European Community and United States of America 2006, Council Regulation (EC) No 555/2008; Commission Implementing Regulation Action (EU) 2016/1150). Crisis distillation of wine was an essential way of the European Union (EU) to get rid of surplus production. However, EU supports for crisis distillation wholly ended in 2012. It has now been reintroduced due to the Covid-19 pandemic (Commission Delegated Regulation (EU) 2020; for Italy DM 2020). There is a general perception, widely shared all around the world, that consumption of traditional wine should decrease in favour of partially dealcoholized wine or wine already made with lower alcohol strength (Heux et al. 2006; Ruf 2011; Mueller 2011; Wine Intelligence Report 2016). The global non-alcoholic wine market is estimated to grow at an impressive CAGR of over 7% during the forecast period (2019–2027) and is projected to reach a value pool of over US $ 10 billion (Report FACT4532MR 2020). In this contest, it makes sense to attempt to improve the technology to meet the evolution of the wine sector, considering that wine is a source of a variety (thousands) of useful components: vitamins, proteins, colourants, molecules with a delicious taste and or smell, minerals, and functional chemical compounds capable of preventing widespread and grave diseases.

Not by chance in the old practice wine was used as a therapeutic agent, for wound dressing, as nutritional dietary beverage, antipyretic, purgative and diuretic (Stockley et al. 2012). Since the French Paradox (Renaud and De Longeril 1992), many systematic studies have been undertaken to identify the beneficial properties of the red wine, of its solid extract, and single molecules of this fraction, regarding bioavailability, efficacy and recommended daily intake amount. For example, the solid extract is characterized by antioxidant properties that positively influence in delaying/preventing the onset of cancer and cardiovascular diseases. The main responsible for these properties are the phenolic fraction, trans-Resveratrol, and quercetin. It is worth to emphasize that bioavailability and health effects of wine-derived solid extract do not require the presence of Ethanol, as aqueous and vegetable matrices are as active as wine in facilitating adsorption (Pellegrini et al. 2000; Goldberg et al. 2003; Roy and Lundy 2005; Baur and Pearson 2006; Paixao et al. 2007; Gorelik et al. 2008).

Consequently, the recommended daily intake of beneficial compounds is assumable while avoiding some drawbacks associated with the excessive consumption of Ethanol which is responsible for some alcohol-related diseases, other than for ethical, social and religious contraindications in many countries. Other research works focused into quantify the physical and the sensorial properties of the aroma compounds of the wine; still, others finalized to understand the stability of the wine and its components to the heat and oxidizing agents (Styger et al. 2011). Therefore, it is reasonable to use an increasing portion of the global wine production as raw material (IW) for making a variety of wine derived products for the food and beverage as well as for para-pharmaceutical industry (Alvarez Gaona et al. 2019).

An interesting strategy for wine fractionation assumes that wine is a liquid solution of four pseudo-compounds (water, Ethanol, solid extract, and volatile aromas) which can be separated to the desired degree of purity as final products or as bases for formulating tailored products required, from time to time, by local and niche markets existing all around the world. This work aims to describe and to discuss an advanced fractionation process for the production of these components under optimal operating conditions, avoiding any damage to their sensorial, nutritional and, functional properties.

## Materials and methods

The wine used in this work was a young “Montepulciano d’Abruzzo” taken from Cantina Citra (Abruzzo, Italy) as the one used in previous work (Di Giacomo and Taglieri 2012). However, it was re-characterized to account for seasonal variations. To this purpose the value of the dry solid extract (g/l) was determined by measuring the density value of the dealcoholized wine (ρ_e_), obtained by slow evaporation and re-condensation, using the Tabarié formula:

$$ \uprho_{\text{e}} = 1 +\uprho_{\text{v}} -\uprho_{\text{d}} $$

ρ_v_ is the density of the wine, while ρ_d_ is the density of the alcoholic distillate, both measured at 20 °C. The value of the total solid extract and that of the alcoholic degree of the wine (abv) were obtained using the Reichard tables as a function of ρ_e_. Actually, for determining the alcoholic degree, the wine was previously alkalinized with about 10% of calcium hydrate. The experimental measurement of the densities was made, in triplicate, using an Anton Paar densimeter (DMA 4100 M), with an estimated error of 0.01%. The alcoholic strength and the solid extract were practically the same as before (13.5 ± 0.03% abv and 2.3 ± 0.02% w/w, respectively). The volatile aroma compounds (VAC) were detected and quantified using a Carlo Erba 8000 top series gas chromatograph (CE Instruments, Milan, Italy), equipped with a flame ionization detector, a split–splitless injector, and a Supel-cowax 10 column (60 m × 0.32 mm). The temperature of both the injector and detector was 250 °C, the carrier gas was helium with a flow rate of 0.8 ml/min, and the split ratio was 1:30. Using a reference mixture, a total amount of about 500 ppm of VAC resulted; the maximum error was 5% on 4 replicates measurements.

The reverse osmosis (RO) operates in dialyzing mode using the vegetation water (RVW) coming from the bottom of the distillation column. The RO experiments were done by using a single-stage pilot plant (NIRO-SOAVI) equipped with a commercial spiral wound module (GEA TFC 3838).

The experimental results, regarding optimal operating conditions, plant configuration, operational procedures, and characteristics of raw materials and products have been already described in detail (Di Giacomo and Taglieri 2012). In particular, it results that RO applied at room temperature with a concentration factor (w/w) < 15, wholly rejects the solid extract, as verified by measuring the density (r_e_) of the dealcoholized retentate. At the same time, Ethanol, water, and VAC are insensitive to the membrane barrier.

## Processes and products

### Technologies for wine fractionation

There are many separation technologies for liquid mixtures, and each of them can be applied, in principle, for fractionating IW. However, the selected technology/technologies should meet some requirements to be efficacious in separating the desired products, economically feasible, and sustainable for energy consumption and environmental impact, while preserving nutritional, sensory and functional properties of each chemical compound. Traditionally, wine fractionation was made by evaporation, or by batch and continuous distillation under atmospheric pressure considering the significant difference between the boiling points of the compounds or groups of compounds to be separated. Atmospheric evaporation and distillation are widely applied for making food-grade alcohol or other distillates. However, they are not applicable when the desired product is the aqueous solution of the solid extract, containing a variety of heat-sensitive compounds, in order to avoid the phenomenon of the cooked wine.

For these reasons when, before the end of the 19^th^ century, a German company started developing a process for producing dealcoholized wine, used vacuum distillation, capable of working with safe temperature, close to ambient one. The sensorial properties of the product were enhanced some year later by developing a technique for recovering the volatile aroma compounds, lost during vacuum distillation (USA Patent 1913). Before the end of the 20th century, new technologies become available at industrial scale for fractionating liquid mixtures and subsequently legalized for application in the food and beverage industry, including the oenology practice. Among these, Reverse Osmosis (RO) and Supercritical Carbon Dioxide Extraction (SCE) appear useful for fractionating industrial wine (Ruiz-Rodriguex et al. 2012; Schmidtke et al. 2012; Diban et al. 2013; El Rayess and Mietton-Peuchot 2016). Both can operate at a temperature lower than 313 K. Furthermore, a wine fractionation dedicated technology, the so-called Spinning Cone Column (SCC) was developed in the USA and further enhanced and applied at industrial scale in Australia (Pichering 2000; Belisario-Sanchez et al. 2009).

RO is in use at industrial scale for producing alcoholic-free wine (alcoholic strength < 0.2% abv) and dealcoholized wine (alcoholic strength < 0.5% abv). However, as discussed in the next paragraph, special precautions must be taken for making uncontaminated and high-quality products. The authors also evaluated the technical feasibility of producing dealcoholized wine by the SCE, using food-grade carbon dioxide as a solvent. The experimental apparatus used for this purpose was a continuous, automatically controlled, pilot plant described in detail in previous work (Del Re and Di Giacomo 1998). Synthetically, it consists of a three meters toll packed-bed insulated column corresponding to about three theoretical stages (unpublished degree thesis on Chemical Engineering of Pietro Romano 2020). Using a constant flow rate stream of fresh solvent, without recirculation, it resulted that an alcohol rich extract continuously lives from the top of the column. At the same time, the dealcoholized raffinate (< 0.2% abv) lives from the bottom. Optimal values of pressure and temperature, which maximize both loading and selectivity are 10 MPa and 313 K respectively while the solvent to feed ratio was equal to 15, w/w. SCE is not in use at industrial-scale for the fractionation of aqueous solutions of Ethanol and other minor solutes, despite the intense research activity documented in the literature (Martinez de la Ossa et al. 1990; Di Giacomo et al. 1993; Budich and Brunner 2003; Macedo et al. 2008; Ruiz-Rodriguez et al. 2012; Schmidtke et al. 2012). This because of the problems arising when recirculating Carbon Dioxide. At the moment, fractionation of industrial wine is mainly performed by RO, vacuum distillation, and by SCC.

### The ROAD process

**Fig. 1:**
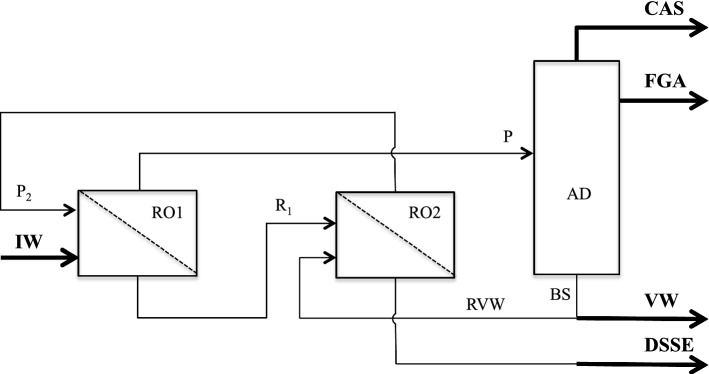
Schematic diagram of the continuous ROAD process equipped with a two-stages countercurrent RO section

Figure 1 shows schematically the ROAD process, i.e., a new method for fractionating industrial wine, obtained by connecting a countercurrent-multistage reverse osmosis section operating in dialyzing mode (RO) with an atmospheric distillation section (AD). In this way, it is possible to reduce the flow rate of washing water and, consequently, to reduce the feed stream of the distillation column. Independently by the number of stages, RO divides IW into two liquid streams: the final retentate (DSSE), and the final permeate (P). The first is made up of the whole amount of the total solid extract, containing all the thermosensitive compounds, dissolved in a concentrated aqueous solution. P is made up mostly of water and Ethanol, plus a tiny amount (ppm) of VAC and is further treated by distillation for separating these three components. Since P does not contain any thermosensitive compound, it is fractionated by the AD, instead of the more energy-intensive vacuum distillation, just avoiding the combined effect of both oxygen and temperature for preserving the integrity of the VAC. Furthermore, heat recovery from the vegetation water (VW) obtained at the bottom of the AD by worming P, enhances the energy efficiency of the whole process.

**Fig. 2:**
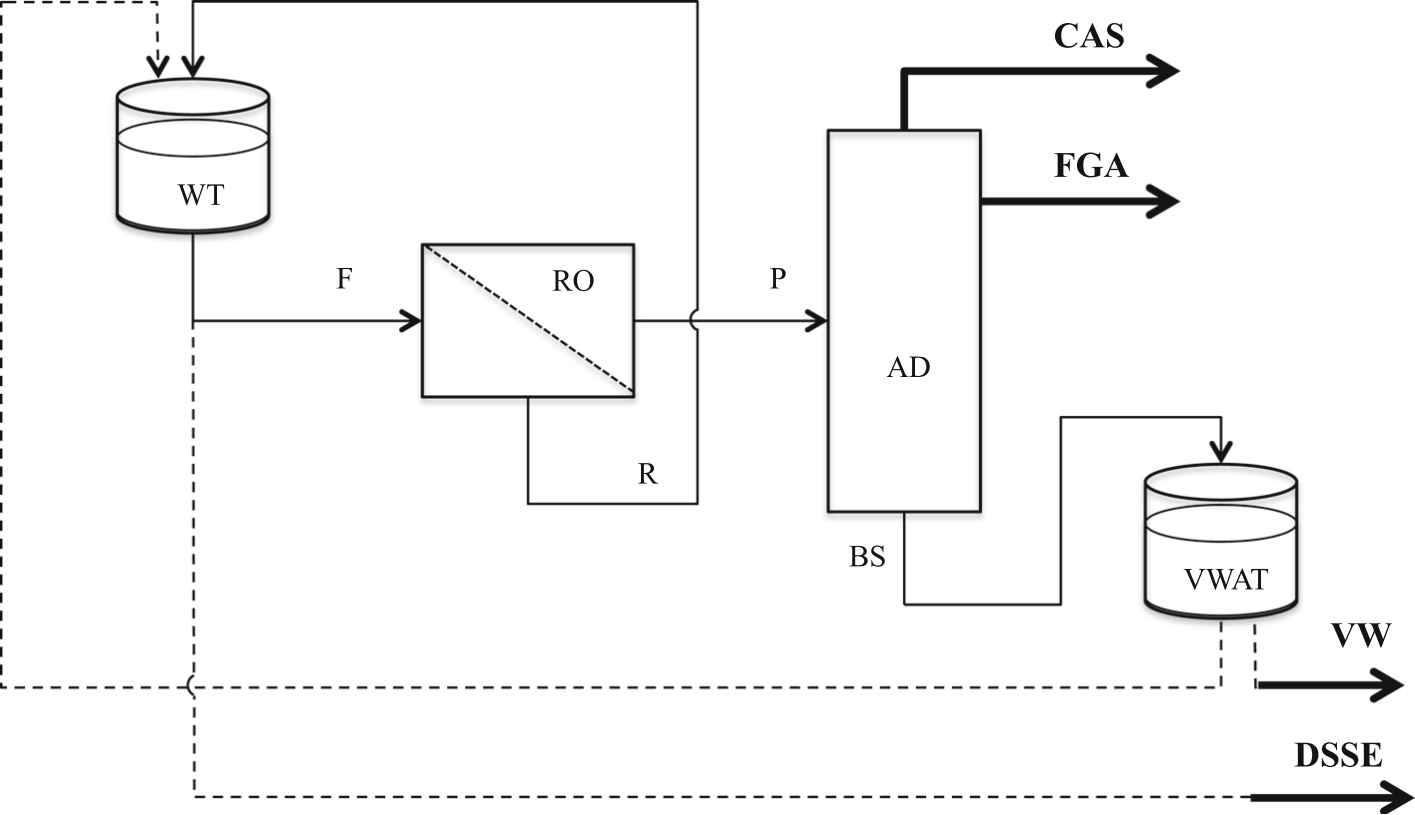
Schematic diagram of the semi-batch ROAD process

An alternative configuration of this process is the one shown in Fig. 2. The first is a continuous process with a counter-current multistage RO section while the second is a semi-batch process with a single-stage RO section. Both give the same products and operate under the same temperature, and pressure conditions using the vegetation water, obtained at the bottom of the AD in the dialyzing RO section. RO operates at ambient temperature and pressure of 3.0–4.0 MPa, while AD operates at ambient pressure and a temperature ranging from about 373 K at the bottom and about 313 K at the top. The choice between these two configurations is mainly related to the potentiality of the plant. Up tens cubic meters of wine per day (local level), the semi-batch one is preferable for lower capital investment as well as for the running costs, other than for the operating flexibility; for processing hundreds cubic meters of IW per day (regional or national level) the continuous one should be preferable. The operating procedure for starting and running a continuous plant is well known; however, referring to the continuous ROAD process one should account for the management of the initial transient that occurs at the beginning, and each time after the backwashing.

## Results and discussion

**Fig. 3:**
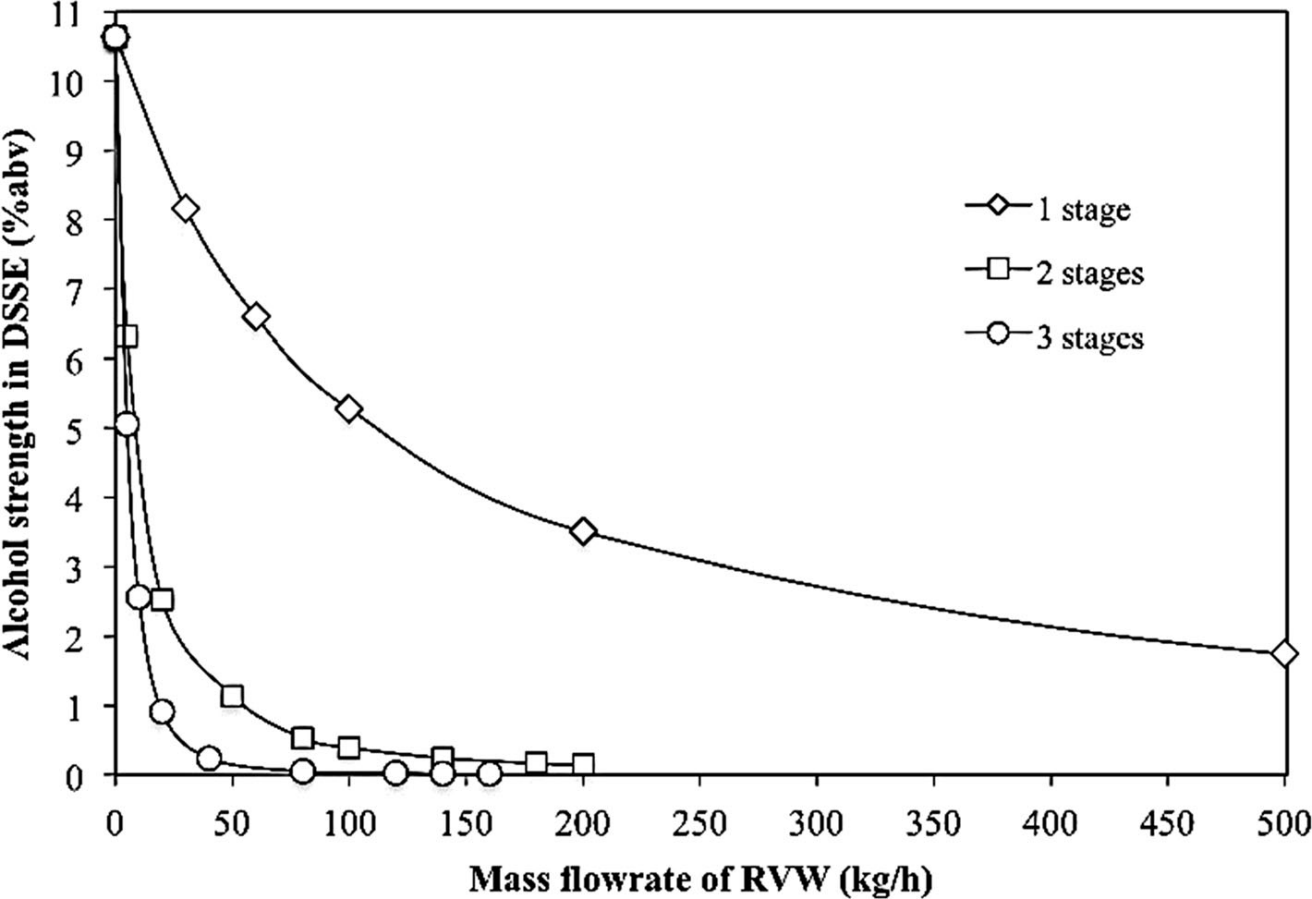
Alcohol concentration in dealcoholized solution of solid extract as function of recirculated vegetation water and number of stages of the countercurrent RO section

As shown in Fig. 2, the mass flow rate of retentate in the individual stages was constant to ensure that the concentration of the solid extract was almost the same and the rejection of the solid solute practically complete. The performance of the process (i.e., the final alcoholic concentration of the dealcoholized aqueous solution of the solid extract) is strongly dependent from both the number of the stages and the flow rate of the recirculating stream of vegetation water (RVW). The results, shown on Fig. 3, allows selecting optimal operating conditions (located on the knee of each curve) concerning the desired degree of the alcoholic strength of the final retentate as well as both processing stability and inexpensiveness.

As can be seen in Fig. 3, using a single-stage RO section the alcoholic strength of DSSE never drops below 2% abv, even when the flow rate of the recycled water is an order of magnitude higher of the water entering with IW. With two stages RO section, the alcoholic strength of DSSE drops to about 0.5% abv when the flow rate of the recycled water is a bit less than the water entering with IW. Finally, with three stages RO section the alcoholic strength of DSSE drops to less than 0.2% abv by recycling about 25% of the water entering with IW.

Table 1 reports the results of the material balance for the continuous process schematically shown in Fig. 1.

**Table 1:** Material balances for the continuous ROAD process

Stream			Stream		
	Mass flowrate (kg/h)	100		Mass flowrate (kg/h)	10
	Water (% w/w)	87		Water (% w/w)	76.6
IW	EtOH (% abv)	13.5 (10.7% w/w)	DSSE	EtOH (% abv)	0.5 (0.4% w/w)
	SE (% w/w)	2.3		SE (% w/w)	23
	VAC (ppm)	500		VAC (ppm)	–
	Mass flowrate (kg/h)	170		Mass flowrate (kg/h)	159
	Water (% w/w)	93.7		Water (% w/w)	100
P	EtOH (% abv)	8 (6.3% w/w)	BS	EtOH (% abv)	–
	SE (% w/w)	–		SE (% w/w)	–
	VAC (ppm)	500		VAC (ppm)	–
	Mass flowrate (kg/h)	10		Mass flowrate (kg/h)	79
	Water (% w/w)	72.2		Water (% w/w)	100
R_1_	EtOH (% abv)	6 (4.8% w/w)	VW	EtOH (% abv)	–
	SE (% w/w)	23		SE (% w/w)	–
	VAC (ppm)	20		VAC (ppm)	–
	Mass flowrate (kg/h)	80		Mass flowrate (kg/h)	0.2
	Water (% w/w)	100		Water (% w/w)	–
RVW	EtOH (% abv)	–	CAS	EtOH (% w/w)	80
	SE (% w/w)	–		SE (% w/w)	–
	VAC (ppm)	–		VAC (%w/w)	20
	Mass flowrate (kg/h)	80		Mass flowrate (kg/h)	11
	Water (% w/w)	99.5		Water (% w/w)	5
P_2_	EtOH (% abv)	0.6 (0.5% w/w)	FGA	EtOH (% w/w)	95
	SE (% w/w)	–		SE (% w/w)	–
	VAC (ppm)	20		VAC (ppm)	–

The required energy for a unit amount of dealcoholized wine by vacuum distillation was calculated by Aspen Plus V10 process simulator in 220 kcal/kg. For comparison, it was also calculated for the continuous ROAD process under optimal operating conditions with two and three RO stages finding, 188 and 183 kcal/kg, respectively. As can be seen, the ROAD process which makes use of countercurrent multistage RO section in dialyzing mode allows saving more than 15% of energy. Furthermore, with the ROAD process, it is possible to separate the four pseudo-components of IW while the vacuum distillation gives only two products. A Similar consideration holds for the SCC technology characterized by an energy consumption about 15% higher than vacuum distillation. Atmospheric distillation is not applicable while RO alone is not comparable because it produces a very dilute alcoholic solution which must be distillated to recover the Ethanol.

When considering the semi-batch ROAD process, the first observation is that there is not an initial transient; furthermore, using two single-stage RO sections that operate alternately, the process becomes practically continuous thus avoiding problems to the regular operation of the AD. A production cycle, based on the semi-batch ROAD process includes two or more steps. The whole amount of VW collected in the corresponding tank (VWAT) at the end of each step, serves for performing the next step. The production cycle starts by charging the washing tank (WT) with the wine to be fractionated, which, in turn, is continuously fed at a constant flow rate (F) to the RO section. Here F is divided into two liquid streams: the Retentate (R), and the Permeate (P). R is made of the whole amount of the total solid extract, containing all the thermosensitive compounds, dissolved in an aqueous solution of Ethanol having the same composition, on solute free-basis than in F; R continuously recycles back to the washing tank (WT). P is made up mostly of water and Ethanol, in the same proportion as they are in the F, plus a tiny amount (ppm) of VAC. Therefore, since P does not contain any thermosensitive compounds, it can be fractionated by the AD, instead of the more energy-intensive vacuum distillation, just avoiding the combined effect of both oxygen and temperature for preserving the integrity of the VAC. The first step ends when the volume of the solution contained in the WT is ten times lower than the initial one. The second step starts by transferring to the washing tank the whole amount of water accumulated in the VW tank during the first step.

The resulting solution is afresh reprocessed by RO using the same procedure as in the first step, i.e. until the volume of the liquid solution in the WT is equal to that was in there at the end of the first step. A third step could reduce further the concentration of Ethanol in the final solution of the solid extract.

As can be seen in Table 2, the alcoholic strength of this solution becomes very low already at the end of the second step.

**Table 2:** Material balances for the semi-batch ROAD process

Tank	Liquid volume and concentrations	1st step		2nd step		3rd step	
		Start	End	Start	End	Start	End
	V (L)	100	10	89.6	10	88.8	10
	Water (% w/w)	87	68.6	96.5	76.3	97.3	76.9
WT	EtOH (% abv)	13.5	10.6	1.19	0.94	0.13	0.09
	SE (% w/w)	2.3	23	2.6	23	2.6	23
	VAC (ppm)	500	55	6.1	0.6	–	–
	V (L)	–	79.6	–	78.8	–	78.7
	Water (% w/w)	–	100	–	100	–	100
VWAT	EtOH (% abv)	–	–	–	–	–	–
	SE (% w/w)	–	–	–	–	–	–
	VAC (ppm)	–	–	–	–	–	–

At the end of a production cycle, one can collect each final fraction into the corresponding storage tank, while refilling WT by wine for starting a new production cycle. Recombining these fractions allows the production of a wide variety of wine-derived custom products. Table 3 shows the volume and the concentrations of the four purified products of the semi-batch ROAD process.

**Table 3:** Volume and concentration of the four pseudo components in the corresponding accumulation vessels at the beginning of the semi-batch 2nd and 3rd processing cycle

Volume and concentrations	Pseudo component							
	2nd cycle				3rd cycle			
	VW	DSSE	FGA	CAS	VW	DSSE	FGA	CAS
V (L)	78.8	10	10.8	0.26	78.7	10	11	0.27
Water (% w/w)	100	76.3	5	–	100	76.9	5	–
EtOH (% w/w)	–	0.7	95	80	–	0.09	95	80
SE (% w/w)	–	23	–	–	–	23	–	–
VAC (% w/w)	–	0.6 ppm	–	20	–	–	–	20

## Conclusion

The proposed ROAD process allows fractionating the industrial wine into its four main pseudo-components, namely: vegetation water, concentrated aqueous solution of the solid extract, food-grade Ethanol, and concentrated alcoholic solution of volatile aroma compounds. It can operate in both continuous and semi-batch mode, according to the potentiality of the plant.

Thanks to a multi-stage reverse osmosis section operating at room temperature, the ROAD process: (a) does not require the addition of any foreign substances, thus avoiding any contamination; (b) it can avoid any organoleptic and functional damage to thermolabile compounds, wholly confined into the concentrated aqueous solution of the solid extract.

Since the Road process results from the interconnection of two fractionation sections operating at quite a different temperature level, it is possible to recover most of the thermal energy of the products streams produced by distillation, for preheating the feed coming from the reverse osmosis section. Consequently, the ROAD process is significantly less energy-intensive of the vacuum distillation and the spinning cone column.

The four products obtainable by applying the ROAD process to the industrial wine are used as such, as bases for similar products by minimal further processing (e.g., freeze-drying of the concentrated aqueous solution of the solid extract) or as an ingredient, mainly for the food and the beverage industry.

However, the most exciting way of using these products is the recombination of two or more of them to make, time by time, a large variety of wine-based products in order to satisfy the requirements of many niche markets already existing, or emerging, all around the world. Some significative examples of already marketable products are: alcoholic-free wine, partially dealcoholized wine with specified alcoholic strength, dyes, healthy products, food-grade alcohol and spirits, and aromas.

## Abbreviations

AD: Atmospheric distillation
BS: Bottom stream
CAS: Concentrated aroma solution
DSSE: Dealcoholized solution of solid extract
EtOH: Ethanol
F: Feed
FGA: Food-grade alcohol
IW: Industrial wine
P: Permeate
P2: Permeate produced by RO2
R: Retentate
RO: Reverse osmosis section
RO1: Stage 1 of the multistage reverse osmosis section
RO2: Stage 2 of the multistage reverse osmosis section
ROAD: Reverse osmosis integrated with atmospheric distillation
R1: Retentate of RO1
RVW: Recirculated vegetation water
SCC: Spinning cone column
SCE: Supercritical carbon dioxide extraction
SE: Solid extract
V: Volume of the liquid
VAC: Volatile aroma compounds
VW: Vegetation water
VWAT: Vegetation water accumulation tank
WT: Washing tank
w/w: Weight fraction
abv: Alcohol by volume
ppm: Parts per million
